# Outcomes of patients with severe and critical COVID-19 treated with dexamethasone: a prospective cohort study

**DOI:** 10.1080/22221751.2021.2011619

**Published:** 2021-12-21

**Authors:** Bernardo A. Martinez-Guerra, Maria F. Gonzalez-Lara, Carla M. Roman-Montes, Karla M. Tamez-Torres, Francisco E. Dardón-Fierro, Sandra Rajme-Lopez, Carla Medrano-Borromeo, Alejandra Martínez-Valenzuela, Edgar Ortiz-Brizuela, Jose Sifuentes-Osornio, Alfredo Ponce-de-Leon

**Affiliations:** a Department of Infectious Diseases, Instituto Nacional de Ciencias Médicas y Nutrición Salvador Zubirán, Mexico City, Mexico; b Clinical Microbiology Laboratory, Department of Infectious Diseases, Instituto Nacional de Ciencias Médicas y Nutrición Salvador Zubirán, Mexico City, Mexico; c Department of Medicine, Instituto Nacional de Ciencias Médicas y Nutrición Salvador Zubirán, Mexico City, Mexico

**Keywords:** Dexamethasone, corticosteroids, COVID-19, SARS-CoV-2, Mexico

## Abstract

Dexamethasone implementation for COVID-19 management represented a milestone but data regarding its impact and safety have not been consistently reproduced. We aimed to evaluate in-hospital mortality before and after the implementation of corticosteroid treatment (CS-T) for severe and critical COVID-19. We conducted a cohort study that included patients admitted with severe and critical COVID-19. The primary outcome was death during hospitalization. Secondary outcomes included the length of stay (LOS), need for invasive mechanical ventilation (IMV), time to IMV initiation, IMV duration, and development of hospital-acquired infections (HAIs). Bivariate, multivariate, and propensity-score matching analysis were performed. Among 1540 patients, 688 (45%) received CS-T. Death was less frequent in the CS-T group (18 vs 31%, *p* < .01). Among patients on IMV, death was also less frequent in the CS-T group (25 vs 55%, *p* < .01). The median time to IMV was longer in the CS-T group (5 vs 3 days, *p* < .01). HAIs occurred more frequently in the CS-T group (20 vs 10%, *p* < .01). LOS, IMV, and IMV duration were similar between groups. Multivariate analysis revealed an independent association between CS-T and lower mortality (aOR 0.26, 95% CI 0.19–0.36, *p* < .001). Propensity-score matching analysis revealed that CS-T was independently associated with lower mortality (aOR 0.33, 95% CI 0.22–0.50, *p* < .01). Treatment with corticosteroids was associated with reduced in-hospital mortality among patients with severe and critical COVID-19, including those on IMV.

## Introduction

Since the early months of the SARS-CoV-2 pandemic, the fact that severe forms of COVID-19 are associated with systemic inflammation [1,2] prompted great efforts to evaluate the effect of numerous anti-inflammatory and immunomodulating therapies [3]. The broad anti-inflammatory effect of steroids in COVID-19 has been evaluated in numerous trials [4–11]. Fortunately, the RECOVERY trial showed decreased mortality with dexamethasone in hypoxaemic COVID-19 patients [4]. Dexamethasone has also been associated with increased ventilator-free days among critically ill patients [7]. A meta-analysis, including the RECOVERY [4] and six other clinical trials, concluded that the administration of systemic corticosteroids was associated with lower 28-day mortality [12]. Updated guidelines recommend the use of dexamethasone in hypoxaemic patients with COVID-19 [13–15]. The implementation of dexamethasone as the standard of care represents a milestone in the rapidly evolving therapeutic strategies for COVID-19. Corticosteroid treatment (CS-T) is the only proven intervention to reduce COVID-19-associated mortality, but such results have not been consistent across trials [4–11]. Likewise, data regarding secondary infections have not been consistently evaluated and are still lacking. As the number of cases continues to increase, COVID-19 remains a major issue in global health. As of 31 October 2021, more than 246 million cases of confirmed COVID-19 and nearly 5 million deaths have been reported by the World Health Organization [16]. We conducted a cohort study to evaluate in-hospital mortality before and after the implementation of CS-T for severe and critical COVID-19.

## Materials and methods

### Patients and settings

We conducted a prospective cohort study in a COVID-19 reference centre in Mexico City. Our centre was converted into a COVID-19 dedicated facility on 16 March 2020. Data of all consecutive patients admitted with a positive SARS-CoV-2 real-time polymerase chain reaction (RT-PCR) between 18 March and 9 November were prospectively registered using the electronic medical record. Patients with severe and critical COVID-19 were included. According to previous definitions, a case was considered severe when SpO_2_ was <93%, PaO_2_/FiO_2_ ratio < 300, respiratory rate ≥ 30 breaths per minute, or ≥50% lung involvement was seen in chest CT; a case was considered critical when either shock, invasive mechanical ventilation (IMV), or multi-organ failure were present [17]. According to institutional protocols, all patients underwent SARS-CoV-2 RT-PCR testing on nasopharyngeal swab samples. Nucleic acid extraction was performed using NucliSens easyMAG system (bioMérieux, Boxtel, The Netherlands) and RT-PCR was processed on Applied Biosystems 7500 thermocycler (Foster City, CA, USA) according to specifications described elsewhere [18]. Each patient was followed-up from admission to death or discharge. The primary outcome was in-hospital death. Secondary outcomes included length of stay (LOS), IMV during follow-up, time to IMV initiation, days on IMV, and development of culture-proven hospital-acquired infection (HAI). A HAI was considered after review by an infectious diseases (ID) specialist to ensure it met accepted criteria [19–22]. Patients who were transferred to other facilities before discharge or death, had an LOS < 24 h, or were diagnosed with moderate disease, were excluded. Because of the observational nature of the study, written informed consent was waived. The study was approved by the Institutional Board Review (Ref. number 3333).

### Corticosteroid administration

After 17 June 2020, following the preliminary results of the RECOVERY trial [23], our centre implemented the use of dexamethasone 6 mg QD for up to 10 days for hypoxaemic patients with COVID-19 as the standard of care. Before that date, steroid use for COVID-19 was not standardized. For this study, CS-T was considered when intravenous dexamethasone ≥ 6 mg QD, prednisone ≥ 40 mg QD, or methylprednisolone ≥ 32 mg QD were used for COVID-19 treatment according to a case-by-case decision by the treating physicians. All patients received the standard of care according to available evidence at the time of admission.

### Statistical analysis

A non-probabilistic, consecutive sampling of all admitted patients that fulfilled the inclusion criteria was implemented. As the study planning started in March 2020, before widespread data were available, no prespecified sample size was calculated. As the study progressed, a sample size calculation was made. Considering a known mortality of 30% in the non-treated (previously published local data [24]) and of 23% in the treated group [4], a probability of type I error (*α*) of 0.05, and a statistical power (1−*β*) of 80%, we calculated a sample size of at least 1246 patients. Data were described using mean, standard deviation (SD), median, and interquartile range (IQR) according to variables’ distribution. Comparisons between patients who received CS-T and those who did not (NCS-T) were made using χ^2^, Fisher’s exact test, independent samples *t*-test, and two-sample rank-sum tests. Bivariate analysis to calculate relative risk (RR) and 95% confidence interval (95% CI) of in-hospital death were performed. A multivariate analysis using a multiple logistic regression model including variables with a *p*-value < .2 in bivariate analysis and of biological importance was performed. Additionally, a propensity score (PS) using a matching method was calculated. To estimate the PS, CS-T was regressed in a logistic regression model. Confounding variables that could affect the outcome and treatment selection were included in the model. For the matching process, we used the logit of the PS using ≤0.1 width calipers of the estimated PS SD. A matching ratio of 1:1 and a non-replacement method were used. To assure balance within the matched sample, a comparison between means, variances, and standardized absolute differences was made. A standardized absolute difference < 0.1 was considered for adequate balance. A post-regression analysis within the matched sample was performed. Finally, the average treatment effect was estimated. Two-sided *p*-values < .05 were considered statistically significant. Missing data were not replaced and were reported in the results. STATA version 15.1 (Texas, USA) was used.

## Results

**Figure 1.**
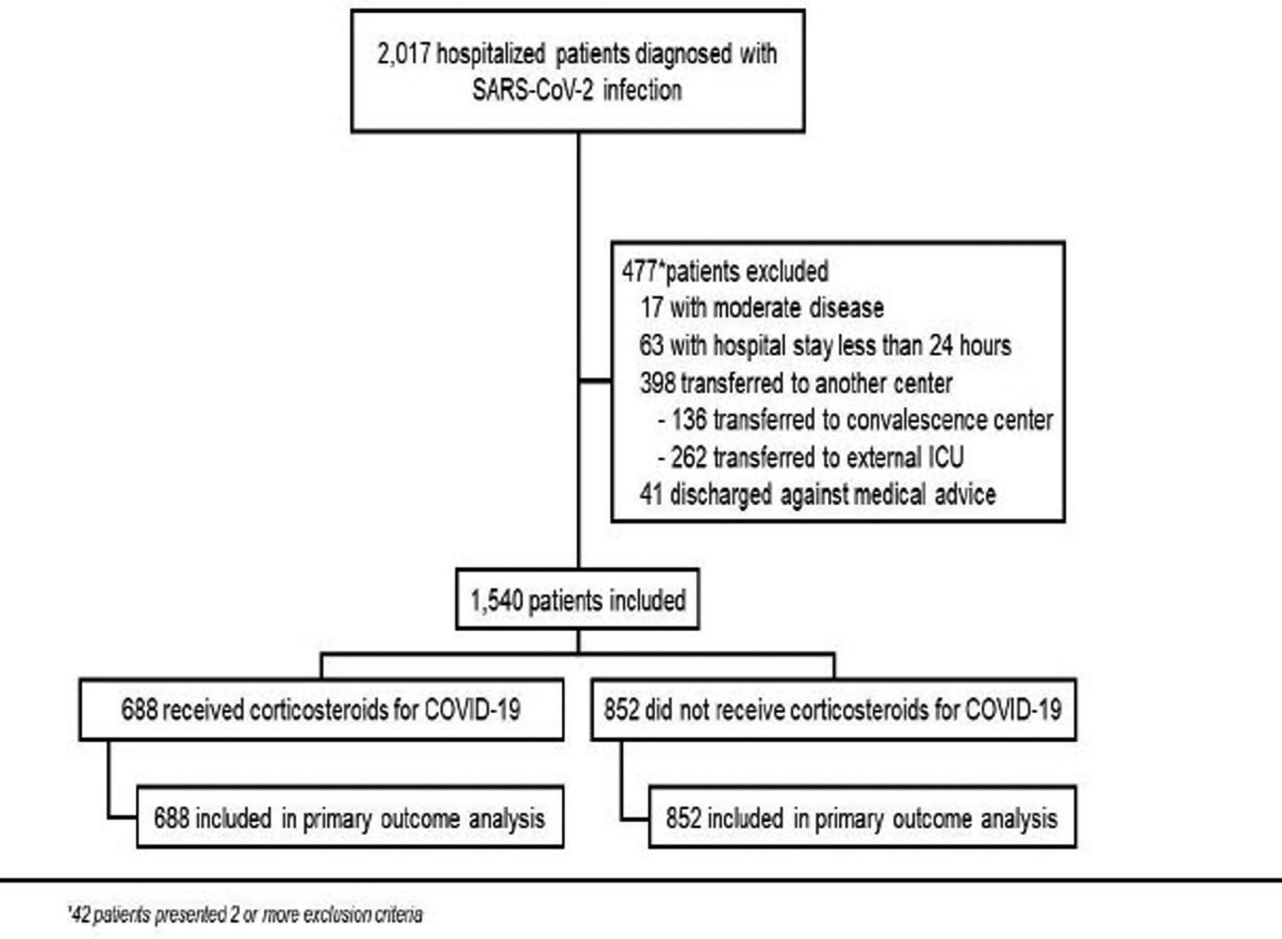
Enrolment and inclusion.

A total of 1540 patients with severe or critical COVID-19 were included, of which 688 (45%) received CS-T (Figure 1). CS-T was more frequent in patients admitted after 16 June [657/743 (88%) vs. 31/797 (2%)]. In the CS-T group, 665 (96.7%) received dexamethasone, 20 (2.9%) methylprednisolone, and 3 (0.4%) prednisone.

The median age was 55 years (IQR 45–65) and 941/1540 (61%) were male. Obesity, type 2 diabetes mellitus (T2DM), and hypertension were present in 681 (44%), 440 (29%), and 528 (34%), respectively. Immunosuppression was present in 87 (6%), being pharmacologic, HIV infection, and malignancy the most common cause in 52/87 (60%), 15/87 (17%), and 15/87 (17%), respectively. The median oxygen saturation, assessed by pulse oximetry (SpO_2_), on admission was 84% (IQR 72–88). The median glucose concentration was 129 mg/dL (IQR 109–183). Except for increased lactate dehydrogenase (LDH) levels in the CS-T group, baseline laboratory results were not different between groups. Patients in the CS-T group had a history of T2DM [225/688 (33%) vs 215/851 (25%), *p* < .01], and a higher ( > 2 points) Charlson comorbidity index score [256/688 (37%) vs 225/851 (26%), *p* < .01], more frequently, and a longer symptom onset on admission [7 days (IQR 6–10) vs 7 days (IQR 5–10), *p* < .05]. Other interventions were different between groups on admission. The use of IMV within 24 h of admission was more frequent in the CS-T [119 (17%) vs 108 (13%), *p* < .05], as well as enrolment in COVID-19 clinical trials [196 (28%) vs 124 (15%), *p* < .01]. Treatment with empiric antibiotics, chloroquine/hydroxychloroquine and tocilizumab, either alone or in combination was more frequent in the NCS-T group [704 (83%) vs 210 (31%), 209 (25%) vs 5 (1%), and 86 (10%) vs 11 (2%), respectively, *p* < .01 for all comparisons] (Table 1).

**Table 1.** Baseline characteristics of the entire cohort.

Characteristic	All patients *n* = 1540 (100%)	Received corticosteroids *n* = 688 (45%)	Did not received corticosteroids *n* = 852 (55%)	*p*-value
Male sex – no. (%)*	941 (61)	418 (61)	523 (61)	.801
Age, years – median (IQR) †	54.5 (45–65)	57 (47–68)	52 (44–63)	<.0001
Obesity – no. (%) *n* = 1537	681 (44)	301 (44) *n* = 688	380 (45) *n* = 849	.692
Diabetes mellitus – no. (%) *n* = 1539	440 (29)	225 (33) *n* = 688	215 (25) *n* = 851	.001
Hypertension – no. (%) *n* = 1359	528 (34)	253 (37) *n* = 688	275 (32) *n* = 851	.067
Chronic obstructive pulmonary disease – no. (%)‡ *n* = 1539	22 (1)	14 (2) *n* = 688	8 (1) *n* = 851	.085
Immunosuppression – no. (%)‡ *n* = 1538	87 (6)	36 (5) *n* = 687	51 (6) *n* = 851	.525
Cardiovascular disease – no. (%)‡ *n* = 1538	86 (6)	46 (7) *n* = 688	40 (5) *n* = 850	.095
Chronic kidney disease – no. (%)‡ *n* = 1539	51 (3)	23 (3) *n* = 688	28 (3) *n* = 851	1.000
Smoker – no. (%) *n* = 1528	231 (15)	111 (16) *n* = 687	120 (14) *n* = 841	.305
Charlson score > 2 – no. (%) *n* = 1539	481 (31)	256 (37) *n* = 688	225 (26) *n* = 851	<.001
Time from symptom onset to admission, days – median (IQR)	7 (5–10)	7 (5–10)	7 (6–10)	.027
Oxygen saturation, % – median (IQR) *n* = 1510	84 (72–88)	84 (74–87) *n* = 682	84 (70–88) *n* = 828	.0811
Glucose, mg/dL – median (IQR) *n* = 857	129 (109–183)	132 (111–191) *n* = 668	123 (106–175) *n* = 189	.0242
Lymphocyte count, cells/µL – median (IQR) *n* = 1530	741 (524–1033)	727 (525–1049) *n* = 684	750 (521–1029) *n* = 846	.7740
C-reactive protein, mg/dL – median (IQR) *n* = 1498	14.7 (8.1–22.1)	14.5 (8.2–21.2) *n* = 671	14.9 (7.8–22.9) *n* = 827	.3081
Ferritin, ng/mL – median (IQR) *n* = 1487	573 (297–1001)	534 (295–921) *n* = 668	616 (311–1069) *n* = 819	.0517
Lactate dehydrogenase, U/L – median (IQR) *n* = 1482	361 (282–475)	342 (266–433) *n* = 676	380 (298–506) *n* = 806	<.0001
D-dimer, ng/mL – median (IQR) *n* = 1500	789 (496–1258)	762 (488–1250) *n* = 678	822 (510–1264) *n* = 822	.1311
SpO_2_/FiO_2_ ratio – median (IQR) *n* = 1492	199 (125–260)	198 (131–259) *n* = 670	201 (118–262) *n* = 822	.2911
Multilobe involvement in CT-Scan – no. (%) *n* = 1538	1530 (99)	687 (100) *n* = 688	843 (99) *n* = 850	.082
ICU admission upon arrival – no. (%)	141 (9)	59 (9)	82 (10)	.478
Use of mechanical ventilation during the first 24 h – no. (%)	227 (15)	119 (17)	108 (13)	.011
Empiric antibiotic treatment – no. (%)	914 (59)	210 (31)	704 (83)	<.001
Tocilizumab - no. (%)‡	97 (6)	11 (2)	86 (10)	<.001
Participation in a clinical trial – no. (%)	320 (21)	196 (28)	124 (15)	<.001

FiO_2_, fraction of inspired oxygen; ICU, intensive care unit; IQR, interquartile range.

*Unless otherwise specified, dichotomous variables were compared using χ^2^.

† Quantitative variables were compared using two-sample rank-sum tests.

‡ Dichotomous variables were compared using Fisher’s exact test.

### Outcomes

**Figure 2.**
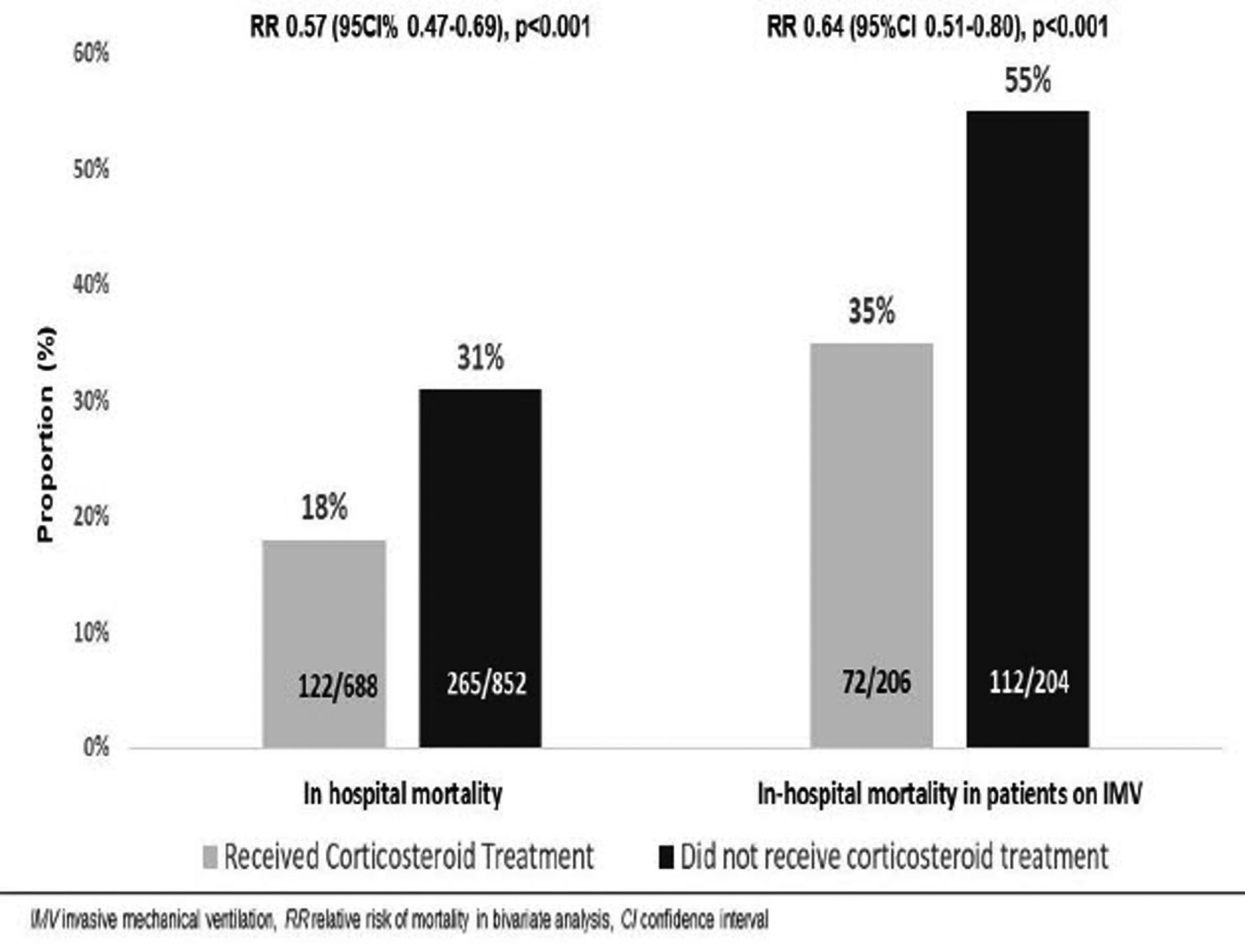
In-hospital mortality.

Outcomes are described in Table 2. In-hospital death occurred in 122/688 (18%) patients in the CS-T group and 265/852 (31%) in the NCS-T group (*p* < .01). Among 410 patients who received IMV, 72/206 (25%) patients in the CS-T group and 112/204 (55%) in the NCS-T group died (*p* < .01) (Figure 2). Among 1153 survivors, LOS was similar between groups [8 days (IQR 5–16) in the CS-T group vs 7 days (IQR 5-13) in the NCS-T group, *p* = .05]. Although the frequency of IMV during follow-up was similar between groups [87/560 (15%) in the CS-T group vs 96/744 (13%) in the NCS-T group, *p* = .22], the median time from admission to IMV was longer in the CS-T group [5 days (IQR 3–7) vs 3 days (IQR 2–4), *p* < .01] (Figure S1, supplementary material). The median IMV duration was similar between groups [13 days (IQR 8–20) in the CS-T group vs 14 days (IQR 11–20) in the NCS-T group, *p* = .41].

**Table 2.** Outcomes in the entire cohort.

Outcome	All patients *n* = 1540 (100%)	Received corticosteroids *n* = 688 (45%)	Did not receive corticosteroids *n* = 852 (55%)	*p*-value
Death – no. (%)*	387 (25)	122 (18)	265 (31)	<.001
Death in patients on mechanical ventilation – no. (%) *n* = 410	184 (45)	72 *n* = 206 (25)	112 *n* = 204 (55)	<.001
Length of stay in survivors, days – median (IQR)† *n* = 1153	7 (5–14)	8 (5–16) *n* = 566	7 (5–13) *n* = 587	.054
Use of mechanical ventilation during follow-up – no. (%) *n* = 1313	183 (14)	87 (15) *n* = 569	96 (13) *n* = 744	.216
Time from admission to mechanical ventilation, days – median (IQR) *n* = 183	4 (2–5)	5 (3–7)	3 (2–4)	.001
Duration of mechanical ventilation in survivors, days – median (IQR) *n* = 226	13 (9–20)	13 (8–20) n = 134	14 (11–20) *n* = 92	.406
Hospital-acquired infection – no. (%)	221 (14)	139 (20)	82 (10)	<0.001
Hospital-acquired infection in patients on mechanical ventilation – no. (%) *n* = 410	201 (49)	125 (61) *n* = 206	76 (37) *n* = 204	<0.001
Hospital-acquired/ventilation-associated pneumonia – no. (%)	158 (10)	103 (15)	55 (7)	<.001
Bloodstream infection – no. (%)‡	66 (4)	34 (5)	32 (4)	.258
COVID-19-associated pulmonary aspergillosis – no. (%)‡	25 (2)	14 (2)	11 (1)	.311
Candidaemia – no. (%)‡	17 (1)	9 (1)	8 (1)	.625

IQR, interquartile range.

*Unless otherwise specified, dichotomous outcome frequencies were compared using χ^2^.

†Quantitative variables were compared using two-sample rank-sum tests.

‡Dichotomous outcome frequencies were compared using Fisher’s exact test.

**Figure 3.**
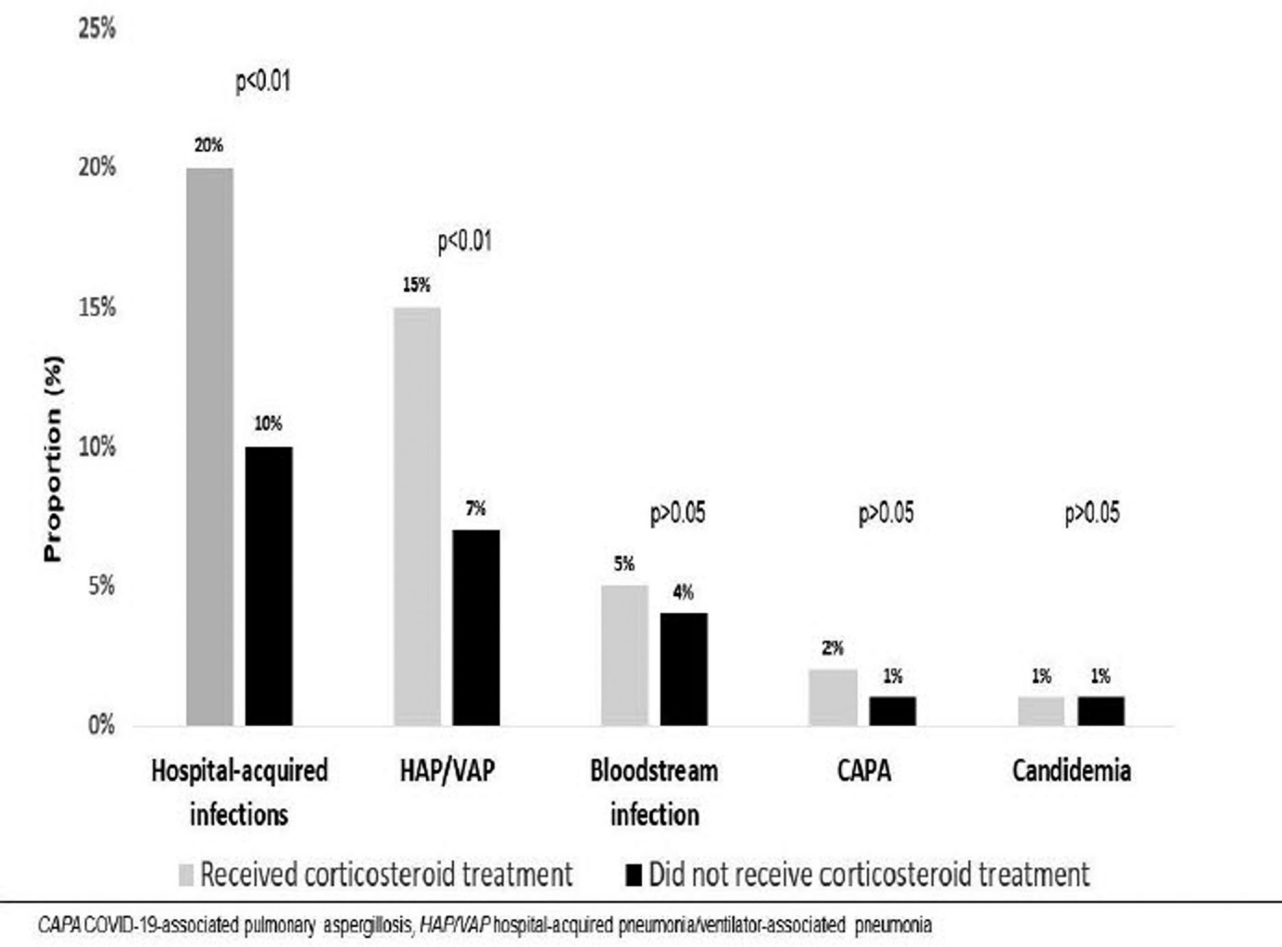
Hospital-acquired infections.

Of note, HAIs occurred more frequently in the CS-T group [139/688 (20%) vs 82/852 (10%), *p* < .01], even after adjusting for IMV status. The most common type of HAI, hospital-acquired/ventilator-associated pneumonia (HAP/VAP), was more frequent in the CS-T group [103/688 (15%) vs 55/852 (7%), *p* < .01]. No differences were seen in bloodstream infections, COVID-19-associated pulmonary aspergillosis (CAPA), or candidaemia (5%, 2%, and 1% in the CS-T group vs 4%, 1%, and 1% in the NCT-S group, respectively) (Figure 3). Microbiological data of 236 episodes of pneumonia and 73 episodes of bloodstream infections are described in Table 3. An episode of blood glucose concentration of 180 mg/dL or greater at day 3 after admission occurred in 65 of 344 patients with available data (19%) in the CS-T group. No major consequences of hyperglycaemia were registered.

**Table 3.** Bacterial microorganisms isolated in infectious episodes in the entire cohort.

Bacterial isolates in 236 pneumonia episodes	No. (%)	Bacterial isolates in 73 bloodstream infection episodes	No. (%)
*Klebsiella* sp.	68 (29)	Coagulase-negative staphylococci	32 (44)
*Enterobacter* sp.	46 (19)	*Enterobacter sp.*	10 (14)
*Pseudomonas* sp.	39 (17)	*Staphylococcus aureus*	5 (7)
*Staphylococcus aureus*	28 (12)	*Klebsiella* sp.	5 (7)
*Escherichia coli*	26 (11)	*Escherichia coli*	3 (4)
*Stenotrophomonas maltophilia*	9 (4)	*Pseudomonas* sp.	2 (3)

### In-hospital mortality

Bivariate analysis showed increased hospital mortality associated with male gender, increasing age, previous comorbidities (T2DM, hypertension, cardiovascular disease, and ischaemic heart disease), higher Charlson comorbidity index score, lower SpO_2_ on admission, baseline lymphocyte count < 800 cells/µL, C-reactive protein (CRP) > 10 mg/dL, ferritin > 500 ng/mL, DHL > 245 U/L, D-dimer > 1000 ng/mL, SpO_2_/FiO_2_ ratio < 300, ICU admission, use of IMV, and development of HAI. CS-T and enrolment in any clinical trial were associated with lower mortality (RR 0.57, 95 CI% 0.47–0.69, and RR 0.29, 95% CI 0.20–0.41, *p* < .01, respectively). Complete results of bivariate analysis are reported in the supplementary material (Table S1). In multivariate logistic regression analysis, CS-T was independently associated with reduced mortality [adjusted odds ratio 0.26 (95% CI 0.19–0.36), *p* < .01]. Enrolment in any clinical trial was also associated with reduced mortality. Male gender, age, lymphocyte count < 800 cells/µL, CRP > 10 mg/dL, D-dimer > 1000 ng/mL, SpO_2_ < 90%, and IMV at any point were independently associated with increased mortality. Development of an HAI was not independently associated with mortality (Table 4).

**Table 4.** Multivariate regression analysis for mortality in the entire cohort.

Variable	aOR (95% CI), *p*
Corticosteroid treatment	0.26 (0.19–0.36), < .001
Male sex	1.57 (1.14–2.14), .005
Age	1.07 (1.06–1.08), < .001
Diabetes mellitus	1.31 (0.96–1.82), .084
Hypertension	0.98 (0.71–1.37), .924
Cardiovascular disease	0.84 (0.47–1.51), .567
Chronic kidney disease	1.57 (0.74–3.33), .242
Baseline lymphocyte count < 800 cells/µL	1.54 (1.13–2.10), .006
Baseline C-reactive protein > 10 mg/dL	3.28 (2.20–4.90), <.001
Baseline D-dimer > 1000 ng/mL	1.63 (1.22–2.19), .001
Baseline oxygen saturation ≤ 90%	1.58 (0.66–3.75), .303
Use of mechanical ventilation	5.66 (3.8–8.43), <.001
Tocilizumab	1.08 (0.62–1.90), .787
Participation in a clinical trial	0.30 (0.19–0.48), <.001
Hospital-acquired infection	0.68 (0.43–1.08), .099
1432 observations, AUC 0.8507, Pseudo-*R*^2^ 0.2847	

Elevated lactate dehydrogenase, troponin I, ferritin, and PaO_2_/FiO_2_ ratio were not included in the model to avoid excessive laboratory abnormalities that are known to be present in patients with severe COVID-19.

ICU admission was not included in the model because in our centre, ICU admission is highly concordant with use of mechanical ventilation.

CI, confidence interval; aOR, adjusted odds ratio.

A PS analysis using data from 1360/1540 (88%) patients was estimated. A total of 968 patients were matched in 484 pairs. The variables included in the model and balance diagnostics are described in Table 5 and Figure S2. Adequate balance within the matched sample was achieved. A logistic regression analysis for in-hospital mortality using the matched sample showed that CS-T was independently associated with lower mortality [adjusted odds ratio of 0.33 (95% CI 0.22–0.50, *p* < .01)] (Table S2). A significant treatment effect was observed within the matched sample regarding in-hospital mortality [82/484 (17%) vs 138/484 (29%), difference −12%, *p* < .01].

**Table 5.** Balance within the matched sample

Variable	Received corticosteroids *n* = 484 (100%)	Did not receive corticosteroids n = 484 (100%)	*p*-value*	Absolute standardized difference
Male sex – no. (%)	293 (61)	292 (60)	.948	0.0042
Age, years – median (IQR)	55 (44–66)	55 (46–65)	.539	0.0400
Obesity – no. (%)	208 (43)	207 (43)	.948	0.0042
Diabetes mellitus – no. (%)	140 (29)	145 (30)	.724	0.0227
Hypertension – no. (%)	163 (34)	171 (35)	.589	0.0348
Chronic obstructive pulmonary disease – no. (%)	6 (1)	5 (1)	.762	0.0195
Immunosuppression – no. (%)	30 (6)	28 (6)	.787	0.0174
Cardiovascular disease – no. (%)	25 (5)	25 (5)	1.00	0.0000
Chronic kidney disease – no. (%)	17 (4)	17 (4)	1.00	0.0000
Oxygen saturation, % – median (IQR)	83 (74–87)	84 (72–88)	.610	0.0327
Time from symptom onset to admission, days – median (IQR)	8 (5–10)	7 (5–10)	.706	0.0242
Lymphocyte count, cells/µL – median (IQR)	714 (519–1001)	753 (523–1023)	.703	0.0245
C-reactive protein, mg/dL – median (IQR)	14.6 (9.0–21.0)	15.1 (7.1–22.3)	.711	0.0238
Ferritin, ng/mL – median (IQR)	560 (303–950)	564 (283–1031)	.808	0.0157
Lactate dehydrogenase, U/L – median (IQR)	363 (275–461)	354 (281–470)	.610	0.0328
D-dimer, ng/mL – median (IQR)	771 (480–1275)	789 (487–1204)	.927	0.0059
Use of mechanical ventilation during the first 24 h – no. (%)	65 (13)	66 (14)	.925	0.0060
Tocilizumab – no. (%)	9 (2)	11 (2)	.651	0.0290
Participation in a clinical trial – no. (%)	80 (17)	99 (20)	.116	0.0999

IQR, interquartile range.

*T-test was used to compare means between groups

## Discussion

This prospective cohort study evaluated the impact of CS-T on hospital mortality of patients with severe and critical COVID-19. We observed that CS-T was associated with decreased in-hospital mortality in patients with severe and critical COVID-19 in the entire cohort and a PS-matched comparative sample. Our results are compatible with the RECOVERY Collaborative Group report [4], which concluded that dexamethasone was associated with a lower mortality in patients receiving supplementary oxygen, including patients on IMV. Our results underscore the importance of inflammatory response in COVID-19-associated mortality. Other factors, such as male gender, increasing age, lower SpO_2_, higher inflammatory markers, and IMV were independently associated with increased in-hospital mortality after multivariate analysis. Such factors have been previously associated with mortality [24–27]. In our study, toclilizumab was not associated with a lower mortality; contradictory results have been reported regarding tocilizumab treatment for COVID-19 [28–31].

Several baseline differences, such as prevalence of T2DM, time from symptom onset to admission, LDH concentration, enrolment in COVID-19-related clinical trials, and IMV initiation within 24 h after admission, were noted between the CS-T and the NCS-T groups. Even though time from symptom onset to admission showed a statistical difference, the difference is not clinically significant.

To minimize confounders and bias, a PS matching analysis was performed. After ensuring an adequate balance within the PS-matched sample, CS-T remained independently associated with a lower mortality risk and a significant treatment effect was observed. Of note, treatment with empiric antibiotics and chloroquine/hydroxychloroquine were not included in the PS model because reports have shown that they have no beneficial impact in COVID-19-associated outcomes [32–36]. The differences in empiric antimicrobial use and chloroquine/hydroxychloroquine may reflect the continuous learning process and standard treatment changes according to the rapidly evolving evidence during the pandemic.

In our study, even though CS-T was associated with a longer time from admission to mechanical ventilation, it was not associated with hospital LOS, IMV during follow-up, and IMV duration, as it has been described in previous reports [4–7].

In our study, 20% of the patients that received CS-T developed an HAI, which is similar to previously reported frequencies among COVID-19 patients treated with corticosteroids (21.9–37.7%) [6,7]. An association between CS-T and development of HAIs in COVID-19 patients has been described [37]. In this study, an association between CS-T and development of HAP/VAP, but not bloodstream infection, CAPA, or candidaemia was observed. The development of new infections is a known adverse effect of immunosuppressive therapy [38]. In contrast to recent reports that associated HAIs with a higher mortality in critically ill patients [39,40], the development of HAI was not independently associated with mortality in this cohort. We believe that a prompt ID consult for all patients with COVID-19 along a sound antimicrobial stewardship program may in part explain this finding.

The high prevalence of T2DM, hypertension, and obesity in COVID-19 patients is consistent with reports from our country [41], where such comorbidities are common in the general population [42].

Limitations in our study must be acknowledged. This was an observational cohort study with unbalanced groups so additional PS matching analysis to minimize bias was performed to increase comparability between groups, rendering favourable results in the CS-T group. Before 17 June 2020, corticosteroid use was not systematic regarding the type, dose, and timing, which led to the variability of prescriptions, although this happened in only 5% of the CS-T group. Although hyperglycaemia was frequent, we cannot assess the impact of CS-T because these data were unavailable for analysis in the NCS-T group, since it was not routinely measured. Additionally, data on biochemical markers of bone turnover were not available. Finally, we cannot separate the effect of the learning curve process and the changing standard of care resulting in improved outcomes within the evaluated time frame, in addition to the expected benefit of corticosteroid use. Still, we believe that our study reflects the improved outcomes after the implementation of a standardized CS-T for severe and critical COVID-19. Nevertheless, the search for COVID-19 effective and safe treatments must continue. As new evidence arises, the impact of CS-T for COVID-19 on comorbidities such as diabetes mellitus and bone metabolism disorders must be studied.

In conclusion, treatment with corticosteroids was associated with reduced in-hospital mortality among patients with severe and critical COVID-19, including those on IMV.

## Geolocation information

Mexico City, Mexico.

## Supplementary Material

Supplemental Material

## Data Availability

The data that support the findings of this study are available from the corresponding author, (J.S.-O.), upon reasonable request.
