# Supplementary material for: Outcomes of patients with severe and critical COVID-19 treated with dexamethasone: a prospective cohort study
Source: Emerg Microbes Infect. 2021 Dec 21;11(1):50–9. doi: 10.1080/22221751.2021.2011619 (PMC8725849; doi:10.1080/22221751.2021.2011619)
Supplement: Supplemental Material [file TEMI_A_2011619_SM4047.docx]

**Supplementary material**

**Outcomes of Patients with Severe and Critical COVID-19 Treated with Dexamethasone: a Prospective Cohort Study**

Bernardo A. Martinez-Guerra, Maria F. Gonzalez-Lara**,** Carla M. Roman-Montes^,^ Karla M. Tamez-Torres**,** Francisco E. Dardón-Fierro, Sandra Rajme-Lopez, Carla Medrano-Borromeo, Alejandra Martínez-Valenzuela, Edgar Ortiz-Brizuela, Jose Sifuentes-Osornio, Alfredo Ponce-de-León

**Table S1.** Bivariate analysis for mortality in the entire cohort

|  | RR (95%CI), p |
| --- | --- |
| Male sex | 1.40 (1.16-1.69), 0.0004 |
| Age | 1.05 (1.04-1.06), <0.001 |
| Obesity | 0.92 (0.77-1.09), 0.3420 |
| Diabetes mellitus | 1.42 (1.19-1.69), 0.0001 |
| Hypertension | 1.36 (1.15-162), 0.0005 |
| Chronic obstructive pulmonary disease | 1.46 (0.83-2.55), 0.2219 |
| Asthma | 0.54 (0.19-1.55), 0.2095 |
| Immunosuppression | 1.01 (0.69-1.46), 0.9780 |
| Cardiovascular disease | 1.42 (1.05-1.92), 0.0325 |
| Chronic kidney disease | 1.34 (0.90-2.00), 0.1705 |
| Liver cirrhosis | 1.08 (0.41-2.85), 0.8734 |
| Smoker | 1.04 (0.82-1.32), 0.7295 |
| Charlson score >2 | 2.07 (1.75-2.45), <0.0001 |
| Time from symptom onset to admission | 0.99 (0.96-1.02), 0.587 |
| Baseline oxygen saturation ≤90% | 2.42 (1.25-4.69), 0.0033 |
| Baseline Lymphocyte count <800 cells/uL | 1.83 (0.51-2.22), <0.0001 |
| Baseline C-reactive protein >10 mg/dL | 3.83 (2.81-5.21), <0.0001 |
| Baseline Ferritin >500 ng/mL | 1.48 (1.22-1.79), <0.0001 |
| Baseline Lactate dehydrogenase >245 U/L | 4.86 (2.85-8.30), <0.0001 |
| Baseline D-dimer >1000 ng/mL | 2.08 (1.75-2.48), <0.0001 |
| Baseline SpO2/FiO2 <300 ratio | 2.52 (1.69-3.78), <0.0001 |
| Critical case on admission | 2.16 (1.79-2.61), <0.0001 |
| ICU admission | 2.62 (2.22-3.09), <0.0001 |
| Use of mechanical ventilation | 2.49 (2.11-2.94), <0.0001 |
| Chloroquine/hydroxychloroquine | 0.86 (0.65-1.12), 0.2497 |
| Tocilizumab | 1.30 (0.96-1.76), 0.1092 |
| Participation in a clinical trial | 0.29 (0.20-0.41), <0.0001 |
| Corticosteroid treatment | 0.57 (0.47-0.69), <0.0001 |
| Hospital acquired infection | 1.65 (1.36-2.01), <0.0001 |
| *CI* confidence interval, *RR* relative risk | |

**Table S2.** Factors associated with mortality in multivariate regression analysis in the matched sample

|  | aOR (95%CI), p |
| --- | --- |
| Corticosteroid treatment | 0.33 (0.22-0.50), <0.001 |
| Male sex | 1.94 (1.24-3.05), 0.004 |
| Age | 1.06 (1.04-1.08), <0.001 |
| Obesity | 0.67 (0.44-1.04), 0.072 |
| Diabetes mellitus | 1.30 (0.85-1.98), 0.230 |
| Hypertension | 1.04 (0.67-1.62) |
| Chronic obstructive pulmonary disease | 1.03 (0.24-4.48), 0.970 |
| Immunosuppression | 2.57 (1.20-5.48), 0.015 |
| Cardiovascular disease | 1.46 (0.68-3.14), 0.336 |
| Chronic kidney disease | 0.89 (0.34-2.37), 0.822 |
| Oxygen saturation | 0.95 (0.93-0.97), <0.001 |
| Time from symptom onset to admission | 0.95 (0.90-0.99), 0.030 |
| Lymphocyte count | 1.00 (1.00-1.00), 0.043 |
| C-reactive protein | 1.04 (1.02-1.06), 0.001 |
| Ferritin | 1.00 (1.00-1.00), 0.076 |
| Lactate dehydrogenase | 1.00 (1.00-1.00), <0.001 |
| D-dimer | 1.00 (1.00-1.00), 0.087 |
| Use of mechanical ventilation during the first 24 hours | 1.26 (0.68-2.34), 0.461 |
| Tocilizumab | 0.40 (0.05-3.32), 0.392 |
| Participation in a clinical trial | 0.42 (0.21-0.82), 0.012 |
| *CI* confidence interval*, aOR* adjusted odds ratio | |

| 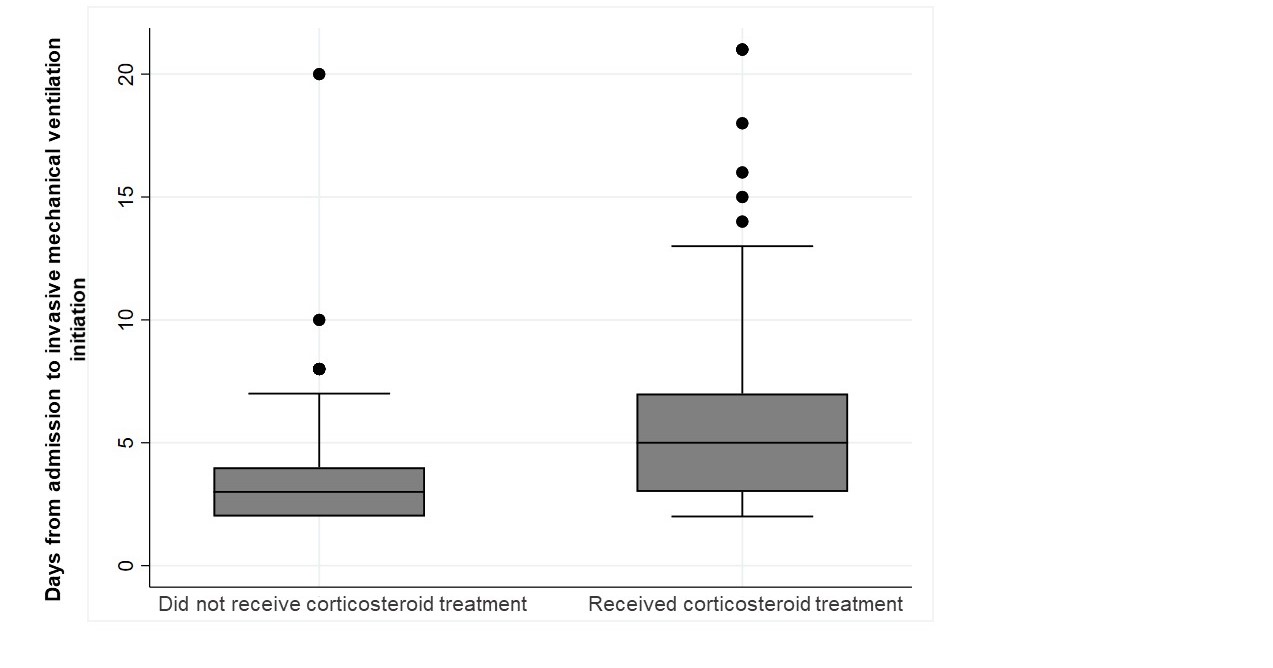 | |
| --- | --- |
| **Figure S1.** Time from admission to invasive mechanical ventilation | |
| 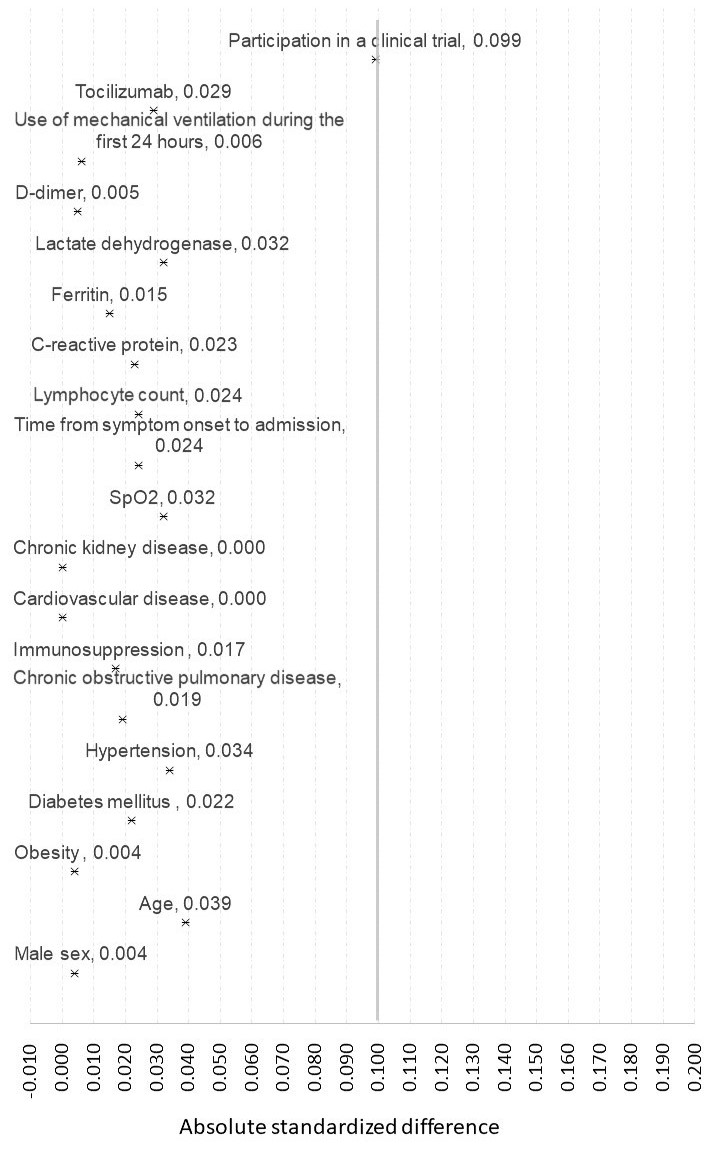 |  |
| **Figure S2**. Absolute standardized difference in the matched sample |  |
